# High-throughput determination of protein affinities using unmodified peptide libraries in nanomolar scale

**DOI:** 10.1016/j.isci.2020.101898

**Published:** 2020-12-07

**Authors:** Clemens Schulte, Vladimir Khayenko, Noah Frieder Nordblom, Franziska Tippel, Violetta Peck, Amit Jean Gupta, Hans Michael Maric

**Affiliations:** 1 Rudolf Virchow Center, Center for Integrative and Translational Bioimaging, University of Wuerzburg, Josef-Schneider-Str. 2, 97080 Wuerzburg, Germany; 2 Nanotemper Technologies GmbH, Flößergasse 4, 81369 Munich, Germany

**Keywords:** Biochemistry Methods, Neuroscience, Biophysical Methods, Biochemistry, Biophysics

## Abstract

Protein-protein interactions (PPIs) are of fundamental importance for our understanding of physiology and pathology. PPIs involving short, linear motifs play a major role in immunological recognition, signaling, and regulation and provide attractive starting points for pharmaceutical intervention. Yet, state-of-the-art protein-peptide affinity determination approaches exhibit limited throughput and sensitivity, often resulting from ligand immobilization, labeling, or synthesis. Here, we introduce a high-throughput method for in-solution analysis of protein-peptide interactions using a phenomenon called temperature related intensity change (TRIC). We use TRIC for the identification and fine-mapping of low- and high-affinity protein interaction sites and the definition of sequence binding requirements. Validation is achieved by microarray-based studies using wild-type and mutated recombinant protein and the native protein within tissue lysates. On-chip neutralization and strong correlation with structural data establish TRIC as a quasi-label-free method to determine binding affinities of unmodified peptide libraries with large dynamic range.

## Highlights

• Protein affinity determination via temperature-related intensity change (TRIC)
• Tracer displacement enables sensitive and quasi-label-free measurements in solution
• Coupling to high-throughput peptide synthesis empowers large-scaled screening
• Largely automated hit identification and affinity determination in nanomolar scale

## Graphical abstract

**Figure:**
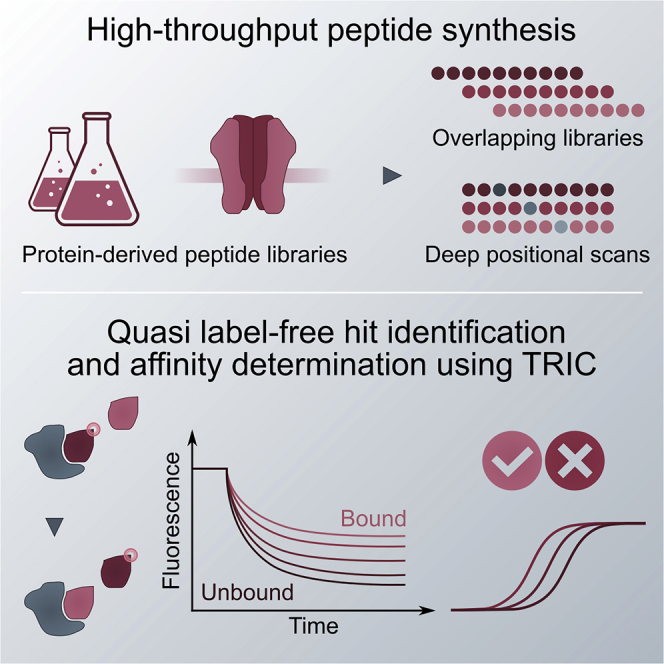

## Introduction

Virtually all cellular processes involve protein-protein interactions (PPIs). A significant fraction of PPIs is dependent on short linear peptides, which are increasingly recognized for their roles in signaling and regulatory networks (Pawson and Nash, 2003) and antibody/antigen recognition (Brennan et al., 2010). Thus, they provide opportunities for therapeutic intervention and valuable starting points for the development of immunological biopharmaceuticals (Fosgerau and Hoffmann, 2015) and the design of PPI modulators (Christensen et al., 2020). Widely applied methods for the characterization and affinity determination of PPIs include peptide and protein microarrays (Lyamichev et al., 2017; Templin et al., 2002), surface plasmon resonance (SPR) (Patching, 2014), biolayer interferometry (BLI) (Sultana and Lee, 2015), isothermal calorimetry (ITC) (Ye and Wu, 2000), fluorescence polarization (FP) (Rooklin et al., 2017), and microscale thermophoresis (MST) (Duhr and Braun, 2006; Wienken et al., 2010). In tandem with high-throughput, nanomolar scale peptide synthesis (Frank, 1992; Sabatino and Papini, 2008), these technologies can be expected to simplify and accelerate protein-peptide interaction analysis and hence the development of pharmaceutical actuators.

Surface-based methods such as SPR, BLI, or array-based techniques provide high sensitivity and throughput. Yet, the required immobilization may affect molecular activity and limit affinity determination. On the other hand, label-free, in-solution affinity determination using ITC, although highly precise, does not allow for exhaustive screenings of large ligand libraries due to the limited sensitivity of calorimetric measurements and the resulting high sample consumption and limited throughput. Here, fluorescent readouts, such as MST and FP, are increasingly employed due to their largely reduced protein requirement, improving assay setup and high predictive value (Jerabek-Willemsen et al., 2011). When set up as displacement assays, they allow for quasi-label-free, in-solution affinity determination. The resulting possibility to use unmodified ligand libraries facilitates broader application. In such a setup, inhibitory constants (K_i_s) are determined by titration of unmodified competitive ligands to labeled protein-peptide complexes. Considerable automatization of sample preparation and their measurements and compatibility with small molecules, fragments, and peptides contributed to the increased application of FP for high-throughput screening (Owicki, 2000). Yet, the FP phenomenon is dependent on ligand and size change upon binding as well as fluorophore and linker characteristics, thus requiring careful assay design (Zhang et al., 2015). In stark contrast, the MST phenomenon is largely independent of molecular weight changes, providing high dynamic range across different assay setups and probe designs (Gupta et al., 2018).

MST setups are highly sensitive; their automatization and miniaturization, however, have been limited by the requirement to track the molecular motion along a temperature gradient in a vessel that prevents turbulent flow (Linke et al., 2015). The dominating physical component of MST is the temperature-related intensity change (TRIC) of fluorescence, which is a measure for the decrease or increase of the fluorescence of a molecule in solution upon heating as a function of time. TRIC allows to detect interactions between target (typically a protein) and ligand with high sensitivity. The nature of the method requires ligand molecules to be non-fluorescent in the investigated red spectral range. Therefore, target molecules are commonly chemically modified with organic fluorophores (Gupta et al., 2018). Here, we explore the use of the Dianthus NT23.PicoDuo (Nanotemper Technologies GmbH) for TRIC measurements in reduced volumes of microtiter plates for affinity determination in unprecedented throughput while maintaining the advantages of conventional MST measurements.

## Results and discussion

We conducted our measurements using the neuronal scaffold protein and master regulator of the inhibitory synapse gephyrin (Tyagarajan and Fritschy, 2014) (geph) and its structurally resolved interaction partners, the glycine receptor (GlyR) β subunit and the γ-aminobutyric acid type A receptor (GABA_A_R) α3 subunit (Maric et al., 2011, 2014; Herweg and Schwarz, 2012). These receptor subunits bind to the E domain of geph (gephE) via a highly conserved linear binding motif. Geph mutations that affect these interactions disrupt the function of distinct synapse types (Hines et al., 2018; Nathanson et al., 2019) and result in neurological disorders (Dejanovic et al., 2014, 2015; Harvey et al., 2008). Low-affinity geph interactions (Brady and Jacob, 2015; Kowalczyk et al., 2013) could not be described for over three decades but were recently resolved thermodynamically (Herweg and Schwarz, 2012; Maric et al., 2011, 2014) and structurally (Kim et al., 2006; Maric et al., 2014) and were exploited to modulate neurotransmission (Maric et al., 2015, 2017; Hines et al., 2018; Kasaragod et al., 2019).

### Peptide arrays enable high-throughput mapping of recombinant and native proteins

**Figure 1:**
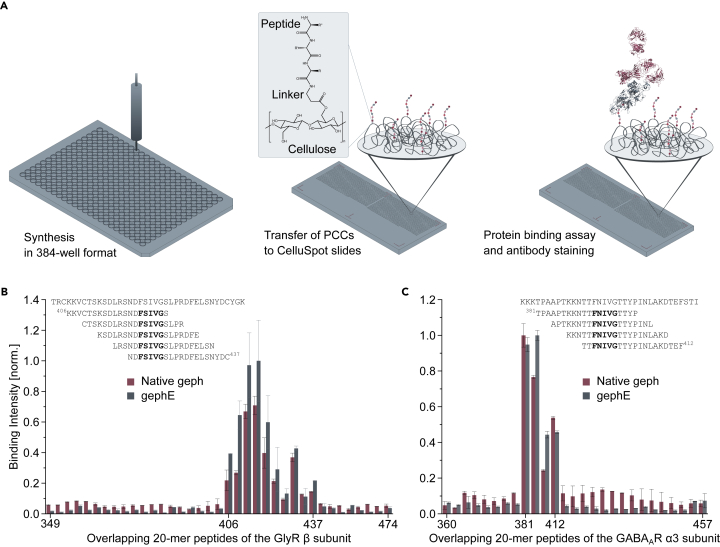
Microarray data Mapping of native and recombinant protein binding sites. (A) Microarray preparation. Up to 4 × 384 peptides are synthesized in parallel on cellulose using standard Fmoc solid-phase peptide synthesis. After sidechain deprotection and dissolution, peptide-cellulose conjugates are printed on coated slides and probed with proteins and their respective antibodies. The intracellular loops between TM 3 and 4 of the GlyR β (B) and the GABA_A_R α3 subunit (C) were displayed via overlapping peptide libraries. Residues delimiting the peptides on-chip are represented on the x axis. Shown are relative binding intensities of native geph (3% and 6% lysate used for GlyR β and GABA_A_R α3, respectively) and gephE (50 nM and 20 nM used for GlyR β and GABA_A_R α3, respectively) for each individual peptide. Amino acid letter codes are displayed for prominent binders. Values are presented as n = 2 with array internal STDEV. Refer to Tables S1 and S2 for peptide sequences.

Peptide microarrays are among the most widely used formats to study antibody-epitope (Price et al., 2012) and peptide-mediated PPIs (Hilpert et al., 2005) in high-throughput. On-chip synthesis (Loeffler et al., 2016) and printable approaches such as Frank's SPOT method (Dikmans et al., 2006; Sereikaite et al., 2019; Hilpert et al., 2007) are among the most common and accessible techniques for microarray production. Here, we used μSPOT (Dikmans et al., 2006) to produce nanomolar scaled peptide stocks (Figure 1A) containing an overlapping GlyR β and GABA_A_R α3 peptide library, which was subsequently printed in microarray format. Array titration with recombinant gephE (Figures 1B, 1C, and S1) recapitulates the structurally resolved (Maric et al., 2014) binding regions of the GlyR β and GABA_A_R α3 subunit (^420^FSIVG^424^ and ^395^FNIVG^399^, respectively). Importantly, the same setup also allows to study native geph within tissue lysates (Figures 1B and 1C). Titrations of the recombinant protein and the lysate yielded strongly correlating binding signals, thus indicating that endogenous levels of the protein are well within the dynamic range of this method and that protein expression and purification may not always be necessary for detailed protein binding studies. To circumvent antibody labeling of the native protein and enable the direct detection of the endogenous protein without further modification *in vitro*, we explored the use of lysates from knock-in mice expressing monomeric red fluorescent protein (mRFP)-geph in μSPOT format. The observed binding pattern showed high correlation to recombinant geph and the immunologically detected native geph but due to peptide autofluorescence and comparably poor quantum yield and photostability of the mRFP, this setup does not appear suitable for accurate affinity determination (Figure S2).

### Validation of high- and low-affinity binding via on-chip neutralization

Major limitations of peptide-based techniques are false-positive and -negative signals resulting from immobilization, orientation, hydrophobicity, aggregation, and charge. Mapped binding sites were validated using known non-binding point-mutated gephE variants (Kim et al., 2006; Maric et al., 2011) (Figure S1B). Validation of specific binding is commonly achieved using binding impaired protein mutants. Yet, because peptide binding sites are often unknown and the prediction of mutations, genetic engineering, and protein repurification is laborious and often not feasible, we here explored alternative means of validation. Namely, *in situ* on-chip peptide neutralization of binding signals (Figure S3). Adding μg amounts of peptides corresponding to the putative binding sites of the GlyRβ (FSIVGSLPRDFELC, **1a**) and the GABA_A_R α3 subunit (FNIVGTTY, **1b**) effectively neutralizes the corresponding interaction (Figure S3). In addition, in line with the lower affinity of the GABA_A_R α3- over the GlyR β-derived peptide, more peptide was needed to neutralize the high-affinity geph-GlyR β interaction (200 μM over 2 μM).

### TRIC allows for sensitive affinity determination in-solution

**Figure 2:**
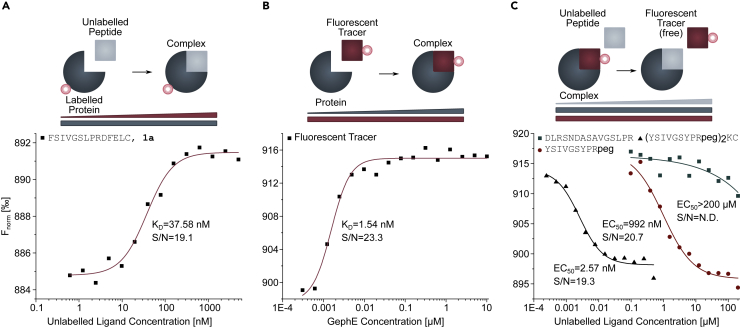
TRIC-based binding assays (A) Red-Maleimide second generation-labeled gephE is titrated with increasing concentrations of an unlabeled, gephE-binding, GlyR β-derived peptide. (B) A fluorescently labeled, dimeric geph-binding peptide is titrated with increasing concentrations of unlabeled gephE. Note that the F_norm_ signal amplitude is increased approximately two-fold in comparison to (A). (C) A complex of unlabeled gephE with the fluorescent tracer is titrated with three unlabeled geph-binding peptides with varying affinity. TRIC measurements recapitulate the known hierarchy of monomeric and dimeric geph binding peptides.

To overcome the inherent caveats of array-based techniques, including immobilization-related artifacts resulting from peptide inactivation or unspecific accumulation of proteins depending on orientation, surface chemistry, and ligand density, we next explored the possibility to measure the same peptide library in-solution using TRIC. Compared with conventional MST setups, the here used TRIC setup allows for reduced sample volumes and is compatible with microtiter plates and thus facilitates higher throughput and automatization (Gupta et al., 2018). Large libraries of unmodified ligands are often analyzed with fluorescently labeled proteins. Here, Alexa 647-labeled gephE exhibited unspecific peptide binding (Figure S1A) as commonly observed. Among the tested alternative labeling strategies, Red-Maleimide second generation (Nanotemper Technologies GmbH)-labeled gephE performed best (Figure 2A). Yet, the observed low-signal amplitude prompted us to explore alternative approaches for titrating unmodified peptides. To this end, we set up a displacement assay inspired by the on-chip neutralization strategy used for validating specific binding in microarray format (Figure S3). Specifically, we identified a high-affinity (K_D_ = 1.54 nM) fluorescently labeled tracer with enhanced TRIC response (Figure 2B). Displacement of this binder yields an inverse TRIC signal and enables the robust affinity determination of unmodified peptides (Figure 2C). Notably, the high-affinity dimeric fluorescent tracer (Maric et al., 2015) provides a large dynamic range for TRIC measurements, even when applied at nanomolar concentrations.

### TRIC in tandem with high-throughput synthesis enables protein-peptide interaction screening

**Figure 3:**
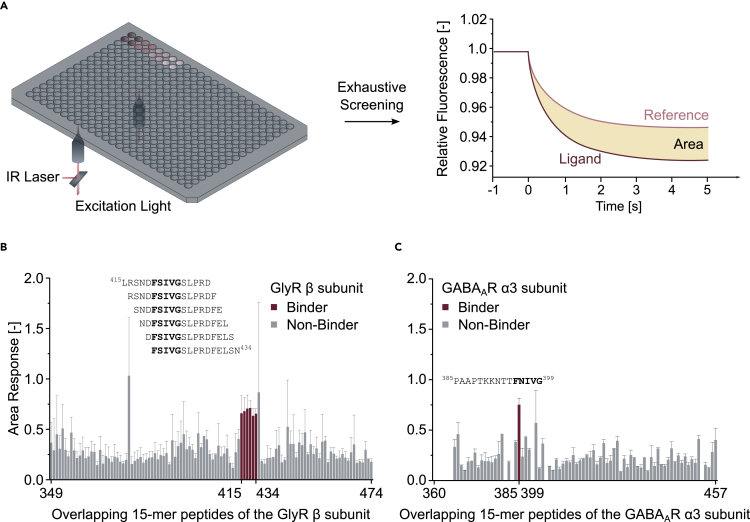
TRIC-based single-dose high-throughput screening TRIC measurement applied for the exhaustive screening of peptide libraries in solution. (A) Schematic representation of a single-dose TRIC setup. Two optical systems operate simultaneously below a 384-microtiter plate for increased throughput at a distance of nine wells. Unmodified peptides are typically screened as a duplicate. Binding is detected by calculating the area between signal traces recorded in presence and absence of the probed ligand. (B and C) Bar graph showing area values for each peptide measured (see Tables S3 and S4 for peptide sequences corresponding to the GlyR β (B) and GABA_A_R α3 (C) subunit respectively and area values for each data point). Residues delimiting the peptides on-chip are represented on the x axis. Hits are highlighted in dark red alongside corresponding peptide sequences with the respective binding motifs in bold. Note that the software gratifyingly classified sequences as binders that harbor the structurally resolved bindings sites ^420^FSIVG^424^ or ^395^FNIVG^399^ (GlyR β and GABA_A_R α3 subunit respectively). Values are presented as n=1-6 with corresponding STDEV if applicable.

We envisioned that the accessible automated peptide synthesis setup that we used for screening in microarray format (Figure 1) could also empower the high-throughput screening of peptide libraries in solution using TRIC. First, a single-dose high-throughput screen was conducted. In this setup, each peptide is incubated with the gephE-tracer complex at a defined concentration in duplicate and subsequently measured. The temperature-dependent fluorescence in each well is monitored as a function of time, followed by the determination of the area between the resulting curves of each peptide and those of the control without competitor (Figure 3A and S5). Peptides exhibiting an area value above a defined threshold (here 0.5) are classified as binders. Peptides are assigned to the binders category after excluding false positives and false negatives. To this end, precipitation and air bubbles are detected by scans of each well in three axial directions. Furthermore, autofluorescence and fluorescence quenching artifacts are identified via additional steady-state fluorescence measurements. The recorded TRIC signal is additionally analyzed by an algorithm that classifies protein/peptide complexes as aggregated.

Here, an overlapping peptide library consisting of 15mer peptides with an offset of one amino acid was prepared to map the benchmark interaction of geph to the GlyR β (Figures 3B and S4A) and GABA_A_R α3 (Figures 3C and S4B) subunit. The software (DI.ScreeningAnalysis Version 1.1.3, November 2020) conducted all described scans, calculations, and assignments automatically to successfully identify the structurally resolved (Maric et al., 2014) binding sites (Figures 3B and 3C), namely, ^420^FSIVG^424^ (GlyR β) and ^395^FNIVG^399^ (GABA_A_R α3) bearing peptides.

### High-throughput in-solution peptide-protein affinity determination using TRIC

**Figure 4:**
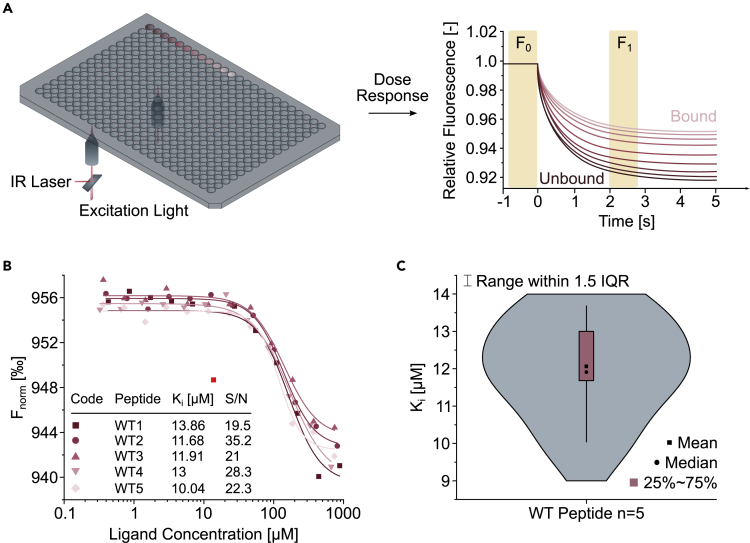
TRIC-based affinity determination (A) Schematic representation of a dose response TRIC setup. Two optical systems operate simultaneously below a 384-microtiter plate for increased throughput at a distance of nine wells. Unmodified peptides are analyzed as a twelve-point dilution series with a dilution factor of two. The resulting TRIC traces are then analyzed for F_norm_ by comparison of the relative fluorescence at F_0_ to F_1_. (B) Comparison of five independent measurements of a GlyR β-derived peptide (^414^DLRSNDFSIVGSLPR^428^) with respective K_i_ and S/N values. The bright red dot was identified as an outliner and excluded from curve fitting. (C) Violin plot showing the mean EC_50_ value of the GlyR β-derived peptides in (B), alongside the mean, median, 25%–75% quantile and range within the 1.5 interquartile range (IQR). The high reproducibility indicates that TRIC-based affinity determination in nanomolar scale is feasible.

Next, we aimed for the affinity determination of screened binders using the same setup and scale that we used for the single dose screen. To assess the reproducibility of the TRIC-based measurements, we subjected five independently synthesized GlyR β-derived peptides (^414^DLRSNDFSIVGSLPR^428^) to a twelve-point dose response measurement (Figure 4A). Here, two-fold increasing concentrations of the unlabeled peptide are incubated with a constant concentration of the gephE-tracer complex. The resulting K_i_ values (mean value: 12.1 ± 1.29 μM) showed low deviation between the median and mean value (Figures 4B and 4C). Thus, substantiating that precise affinity determination in this scale using the described setup is indeed feasible.

### Comparison of microarray- and TRIC-based interaction profiles

**Figure 5:**
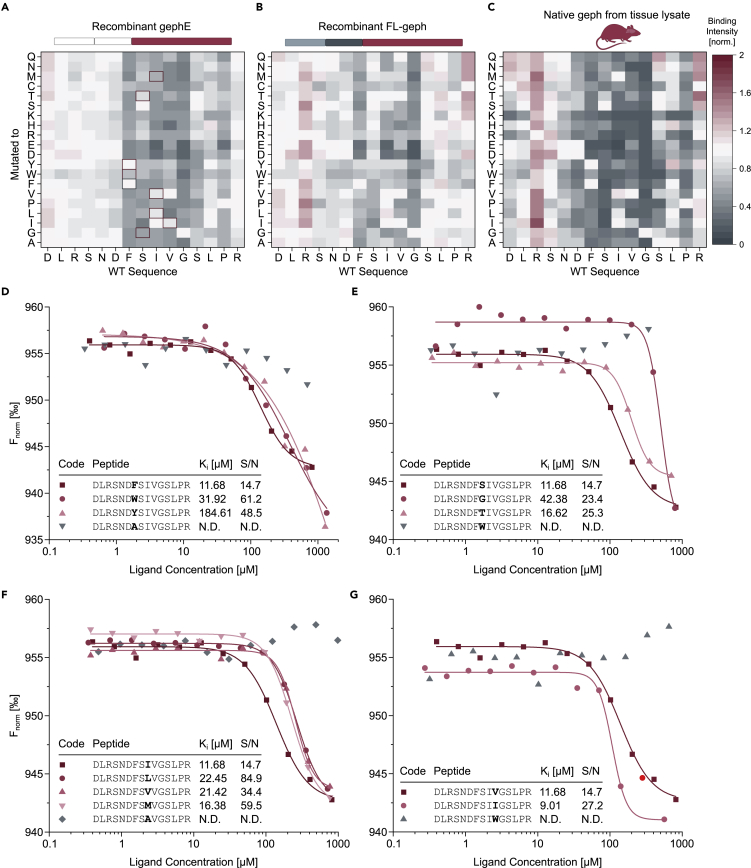
Comparison between binding profiles of GlyR β to gephE, FL-geph, and native Geph in μSPOT and TRIC format (A) A full positional scanning library was probed with recombinant gephE, FL-geph (B), and native geph from tissue lysate (C) in microarray format. Geph domain architectures of the applied recombinant protein or lysate origin are indicated above the heatmaps. Shown are intensity values of point-mutated variants normalized to the corresponding WT sequence (GlyR β ^414^DLRSNDFSIVGSLPR^428^) displayed as a heatmap. Higher spot intensity corresponds to preferential binding, vice versa. In A, brackets corresponding to peptides that exhibited a determinable K_i_ value in TRIC format are bordered in red, whereas a representative WT sequence is bordered in blue. Values are presented as mean of n = 3. Refer to Table S5 for STDEV of each bracket. (D–G) Determined dose responses and derived K_i_ values of peptide variants in TRIC format. The interchanged position (position 420 (D), 421 (E), 422 (F) and 423 (G) of the GlyR β subunit respectively) is highlighted in bold. Peptides with a quantifiable K_i_ value are shown in red, alongside a representative non-binding variant in gray. Corresponding brackets in (A) are bordered in red. Bright red dots were excluded from curve fitting. Refer to Table S6 for starting concentrations of each peptide and Figure S7 for a direct comparison of the binding intensity found in microarray format and K_i_ values determined using TRIC.

Next, we combined TRIC measurements with high-throughput peptide synthesis to facilitate a more precise profiling of protein-peptide interactions. This was achieved by determination of residual affinities for all possible point-mutated variants of the earlier resolved (Maric et al., 2014) benchmark geph—GlyR β and GABA_A_R α3 interaction. First, the identified GlyR β binding motif was subjected to a full positional scan in μSPOT format. Here, each position of the binding sequence is systematically interchanged to define binding requirements and the contribution of each amino acid in the binding sequence. The copyability of μSPOT arrays facilitated the characterization of the isolated binding domain gephE (Figure 5A), full-length geph (FL-geph) (Figure 5B), and native geph from tissue lysate (Figures 5C and S2) by enabling the generation of 5,400 individual data points over 9 arrays. In each of these scans, the highly conserved and thus highly mutation sensitive GlyR β binding motif ^420^FSIVG^424^ was robustly resolved. Thus, validating that protein profiling with molecular resolution can be achieved in this setup even without necessitating expression, purification, and dye-conjugation of the target protein (Figure 5C).

To demonstrate the throughput and accuracy of our new setup, we complemented the microarray data with the corresponding TRIC-based in-solution measurements. To this end, a full positional scan of the GlyR β-derived peptide ^414^DLRSNDFSIVGSLPR^428^ with variations within the ^420^FSIVG^424^ sequence was performed in TRIC-format. Binding affinities of peptide variants were determined by 12-step titrations (Figures 5D–5G, S6, and S7). Direct comparison of the in-solution determined K_i_ values with the relative binding intensities from the microarray (sequences highlighted in red in Figures 5A, 5D–5G, and S7) reveal a strong correlation. Importantly, the obtained affinities are in line with previously determined values and the structurally resolved interaction interface (Maric et al., 2011, 2014; Kim et al., 2006). These data suggest that the here described TRIC setup allows for the definition of protein binding profiles in high throughput at a level of detail that is usually only achieved by laborious and low-throughput PPI affinity determination approaches.

## Discussion

This study defines TRIC as a novel and useful means for the high-throughput in-solution affinity determination of protein-ligand interactions. The setup was used for the rapid screening of 220 unmodified and nanomolar-scaled peptides and the immediate affinity determination of hits and all mutational variants that exhibit residual or enhanced binding. Using a displacement approach, we were able to boost the signal amplitude compared with measurements with a fluorescently labeled protein, while facilitating a quasi-label-free measurement of an unmodified peptide library. The obtained K_i_ values exhibited high reproducibility and correlated well with previously determined values (Maric et al., 2015). The binding data were complemented by conventional mapping and profiling using peptide microarrays, which are commonly employed for the characterization and mapping of protein-peptide interactions (Hilpert et al., 2005; Price et al., 2012). Here, we demonstrated that *ex vivo-*derived native protein within lysate can be used for direct microarray-based profiling studies. To overcome the required peptide immobilization that may introduce systematic biases, we employed TRIC to study the identical peptide libraries and demonstrate its use for screening as well as immediate affinity determination in solution. As such, this approach complements array-based approaches to provide molecular level characterization and furthermore provides a close-to-native measure of protein-peptide interaction strength that is usually only achieved after laborious and often low-throughput biophysical characterization.

In contrast to widely applied FP measurements, TRIC-based approaches provide higher flexibility regarding the properties and linkage of the fluorescent group. This is because only the fluorescent intensity upon temperature change is measured, whereas local mobility of the fluorophore does not influence the readout (Nasir and Jolley, 1999). The here described TRIC setup consumed around 50 ng protein, 2ng of the fluorescent tracer, and up to 700μg of studied peptide variant for a full 12-point dose response measurement. This enables its coupling to our high-throughput synthesis and thus any synthesis approach with the same nanomolar scaling including variants of Frank's highly accessible SPOT synthesis (Frank, 1992) as well as solid cellulose (Dikmans et al., 2006) or polystyrene bead-based (Murray et al., 2005) alternatives. Our approach allowed for synthesis of 768 or 384 peptides in parallel for single-dose measurements and 12-point dose responses, respectively. In addition to analysis of peptide binders that directly compete with the labeled tracer, the here presented setup also allows for the screening and subsequent affinity determination of small molecule and fragment libraries or even proteins and antibodies. Future studies may further emphasize on other applications that would be difficult to tackle with conventional binding assays including the ability of TRIC to detect binding events that do not cause a displacement of the fluorescently labeled tracer but rather result in the formation of a ternary complex. We expect that high-throughput TRIC measurements in combination with similar or alternative ligand library synthesis approaches will greatly accelerate the detailed mapping and characterization of binding sites and thereby help to decipher PPIs and their pharmaceutical targeting.

### Limitations of the study

In the here presented work, we employ a displacement TRIC approach for the characterization of a benchmark PPI, which circumvents protein or ligand labeling and, at the same time, boosts the signal amplitude. Yet, the dynamic range of quantifiable affinities is limited by the affinity of the fluorescently labeled tracer peptide, similar to displacement setups using FP (Christensen et al., 2020). Consequently, to determine the affinity of lower affinity binders, the affinity of the tracer molecule would need to be adjusted accordingly. This will be crucial in fragment screenings, where the affinity determination of low-affinity fragments is of interest to maturate a high-affinity lead compound.

Peptide studies in solution are inherently limited by sequence-dependent aggregation, autofluorescence, and solubility. The here used TRIC setup allows to detect and subsequently exclude aggregation and autofluorescence and further ligand-induced fluorescence quenching by the peptide ligand (see Tables S3 and S4).

To fully exploit the throughput of TRIC measurements, we employed them in tandem with high-throughput μSPOT peptide synthesis followed by cleavage via a Rink-amide moiety. Although this approach is virtually unlimited with regard to possible peptide building blocks, this cleavage will leave peptides as terminal amides as do other commonly applied cleavage methods (Mcbride et al., 2016). Studies of PPIs that rely on interactions with C-terminal extremities, such as PDZ-domains (Christensen et al., 2019), may have to apply alternative cleavage strategies (Fraczyk et al., 2018). Here, we validated μSPOT synthesized peptides by LCMS and HPLC (Table S7) and determined the average purity of 15mers as 61 ± 24% and 47 ± 12% for 20mers. Accordingly, additional universal peptide purification steps could greatly enhance the robustness, sensitivity, and predictive value, especially for peptides exceeding 20 amino acids. Here, we employed preparatively synthesized and purified peptides for on-chip validation of signal specificity. To maintain high throughput, the on-chip neutralization could be adjusted so that peptides could be drawn from the same peptide stocks that are used for array printing.

### Resource availability

#### Lead contact

Further information and requests for resources and reagents should be directed to and will be fulfilled by the Lead Contact, Hans Michael Maric (Hans.Maric@uni-wuerzburg.de).

#### Materials availability

There are restrictions to the availability of the fuorescent tracer due to the lack of an external centralized repository for its distribution and our need to maintain the stock. We are glad to share the fluorescent tracer with reasonable compensation by requestor for its processing and shipping.

#### Data and code availability

The published article includes all datasets generated or analyzed during this study.

## Methods

All methods can be found in the accompanying Tranparent methods.

## Supplemental information

Document S1. Transparent methods, Figures S1–S7, and Tables S1, S2, and S7

Table S3. Overview of overlapping GlyR β peptide library in TRIC, related to Figure 3 Peptide sequences are given with their delimiting residue numbers alongside the Area Response obtained in a displacement setup with Alexa647-labeled NN1D (geph binding peptide) and gephE. Area values presented in gray were not considered due to automatic detection of, e.g., scan anomalies. Observed fluorescence effects (quenching, auto-fluorescence, or quenching) of the peptide are listed. All peptides are categorized as binders (binding detected using a threshold area of 0.5), non-binders (no binding detected), not reproducible (aggregation detected for at least one datapoint), and potential hits. The normalization ID refers to the position of the positive control (unlabeled NN1D), to which the respective TRIC curve was normalized. Signal quality provides a measure for the signal over noise ratio of each measurement.

Table S4. Overview of overlapping GAB_A_AR α3 peptide library in TRIC, related to Figure 3 Peptide sequences are given with their delimiting residue numbers alongside the area response obtained in a TRIC displacement setup with Alexa647-labeled NN1D (geph binding peptide) and gephE. Area values presented in gray were not considered due to automatic detection of, e.g., scan anomalies. Observed fluorescence effects (auto-fluorescence, quenching) and measurement details (scan anomaly, fluorescence too high) of individual data points are listed. All peptides are categorized as binders (binding detected using a threshold area of 0.5), non-binders (no binding detected), not reproducible (determined by internal algorithm), and potential hits. The normalization ID refers to the position of the positive control (unlabeled NN1D), to which the respective TRIC curve was normalized. Signal quality provides a measure for the signal over noise ratio of each measurement.

Table S5. Overview of GlyR β full positional scanning peptide library in μSpot, related to Figure 5 The respective peptide sequence is given for each sequence index alongside the relative binding intensity and STDEV obtained for gephE, Fl-geph, and native geph from mouse brain lysate. Relative binding intensities are given as n = 3 from independent experiments.

Table S6. Overview of GlyR β positional scan peptide library in TRIC, related to Figure 5 The respective peptide sequence is given for each sequence index alongside the molecular weight and maximum concentration (C) used for dose response assays. The obtained EC50 value is given for each peptide.
