# Supplementary material for: High-throughput determination of protein affinities using unmodified peptide libraries in nanomolar scale
Source: iScience. 2020 Dec 7;24(1):101898. doi: 10.1016/j.isci.2020.101898 (PMC7753147; doi:10.1016/j.isci.2020.101898)
Supplement: Document S1. Transparent methods, Figures S1–S7, and Tables S1, S2, and S7 [file mmc1.pdf]

iScience, Volume *24*

Supplemental Information

High-throughput determination of protein affinities using unmodified peptide libraries in nanomolar scale

Clemens Schulte, Vladimir Khayenko, Noah Frieder Nordblom, Franziska Tippel, Violetta Peck, Amit Jean Gupta, and Hans Michael Maric

Supplemental Information

High-throughput Determination of Protein Affinities using Unmodified Peptide Libraries in Nanomolar Scale

Clemens Schulte[1]; Vladimir Khayenko[1]; Noah Frieder Nordblom[1]; Franziska Tippel[2], Violetta Peck[1]; Amit Jean Gupta[2]; Hans Michael Maric[1]*

[1]Rudolf Virchow Center; Center for Integrative and Translational Bioimaging; University of Wuerzburg; Josef-Schneider-Str. 2, Germany, 97080 Wuerzburg, Germany

[2]Nanotemper Technologies GmbH, Flößergasse 4, 81369 Munich, Germany

Transparent Methods

Unless otherwise stated, amino acids and reagents were purchased from either Iris Biotech or Carl Roth. All solvents were purchased from commercial sources and used without further purification.

Automated Solid-Phase Peptide Synthesis

µSPOT peptide arrays(Dikmans et al., 2006) (CelluSpots, Intavis AG, Cologne, Germany) were synthesized using a MultiPep RSi robot (Intavis AG) on in-house produced, acid labile, amino functionalized, cellulose membrane discs containing 9-fluorenylmethyloxycarbonyl-β-alanine (Fmoc-β-Ala) linkers (average loading: 131 nmol/disc – 4 mm diameter). Synthesis was initiated by Fmoc deprotection using 20% piperidine (pip) in dimethylformamide (DMF) followed by washing with DMF and ethanol (EtOH). Peptide chain elongation was achieved using a coupling solution consisting of preactivated amino acids (aas, 0.5 M) with ethyl 2-cyano-2-(hydroxyimino)acetate (oxyma, 1 M) and *N*,*N'*-diisopropylcarbodiimide (DIC, 1 M) in DMF (1:1:1, aa:oxyma:DIC). Couplings were carried out for 3×30 min, followed by capping (4% acetic anhydride in DMF) and washes with DMF and EtOH. Synthesis was finalized by deprotection with 20% pip in DMF (2×4 µL/disc for 10 min each), followed by washing with DMF and EtOH. Dried discs were transferred to 96 deep-well blocks and treated, while shaking, with sidechain deprotection solution, consisting of 90% trifluoracetic acid (TFA), 2% dichloromethane (DCM), 5% H_2O and 3% triisopropylsilane (TIPS) (150 µL/well) for 1.5 h at room temperature (rt). Afterwards, the deprotection solution was removed, and the discs were solubilized overnight (ON) at rt, while shaking, using a solvation mixture containing 88.5% TFA, 4% trifluoromethanesulfonic acid (TFMSA), 5% H_2O and 2.5% TIPS (250 µL/well). The resulting peptide-cellulose conjugates (PCCs) were precipitated with ice-cold ether (0.7 mL/well) and spun down at 2000×g for 10 min at 4 °C, followed by two additional washes of the formed pellet with ice-cold ether. The resulting pellets were dissolved in DMSO (250 µL/well) to give final stocks. PCC solutions were mixed 2:1 with saline-sodium citrate (SSC) buffer (150 mM NaCl, 15 mM trisodium citrate, pH 7.0) and transferred to a 384-well plate. For transfer of the PCC solutions to white coated CelluSpot blank slides (76×26 mm, Intavis AG), a SlideSpotter (Intavis AG) was used. After completion of the printing procedure, slides were left to dry ON.

Peptides used for MST measurements were synthesized using a cleavable amide linker. After synthesis, cellulose disks were transferred to 96 deep-well blocks and treated with

sidechain deprotection solution for 3 hrs at rt under agitation. The solution was subsequently transferred to new 96 deep-well plates and 700 µL ice cold ether were added to each well. After ON precipitation of the peptides at -20 °C, the 96 deep-well blocks were centrifuged at 2,000 xG for 30 min and the supernatant (SN) was discarded. After an additional wash with 700 µL ice cold ether, peptides were solubilized in MST assay buffer consisting of 1×phosphate-buffered saline (PBS; 137 mM NaCl, 2.7 mM KCl, 10 mM Na_2HPO_4, 1.8 mM KH_2PO_4, pH 7.4) with 0.1% Pluronic F-127 (5% solution, Nanotemper Technologies GmbH) and 2 mM reduced L-Glutathione (GSH, Sigma-Aldrich G4251).

Preparative Peptide Synthesis

Standard solid phase peptide synthesis with Fmoc chemistry was applied, shortly, 2-chlorotrityl resin (1.6 mmol/g) was swollen in dry DCM for 30 min., then, the desired aa (1eq) and the orthogonally protected Boc-Gly-OH (1eq) with 2 eq. of dry N,N-Diisopropylethylamine (DIEA) were added to the resin slurry. Boc-Gly-OH was added to reduce resin loading to prevent aggregation of the growing peptide chain. After ON reaction, the resin was capped with MeOH and washed with DCM and DMF. Deprotection and conjugation cycles followed, where 20% pip solution in DMF was used to remove the Fmoc protecting group, and after washes the peptide chain was elongated by adding aa (3eq.) with oxyma (3eq.) and DIC (3eq.). Coupling efficiency was monitored by measuring the absorption of the dibenzofulvene–pip adduct after deprotection. The peptides were cleaved from the resin using a cocktail of 82.5% TFA, 5% phenol, 5% H_2O, 5% thioanisole, 2.5% 1,2-ethanedithiol for 4 h at rt. The peptides were precipitated in ice-cold ether and then purified with high performance liquid chromatography (HPLC) and analysed by liquid chromatography-mass spectrometry (LCMS).

Protein Labeling

The protein construct of gephE (residues 318-736) was generated as previously described(Maric et al., 2014). Labeling with Alexa Fluor™ 647 NHS-Ester (Succinimidylesther) was achieved using a labelling kit (Invitrogen, A37573) according to the manufacturer's instructions. Labeling with Red-Maleimide 2nd generation was achieved using a labelling kit (Nanotemper, MO-L014) according to the manufacturer's instructions.

Preparation of Mouse Tissue Lysates

Whole mouse brains were obtained from C57BL/6J mice at >4 weeks of age and immediately flash frozen in liquid N_2. Tissue of mRFP-gephyrin knock-in animals of 1 year of age (provided by Christian Specht, IBENS, Paris) was processed the same way. Before lysis, whole mouse brains were weighted and cut into four pieces along the coronal and sagittal axis. For preparation of one lysate, two diagonally opposite pieces were transferred into a 1.5 mL reaction tube (Sarsted). Lysis was carried out on ice in 400 µL RIPA lysis buffer (150 mM NaCl, 50 mM Tris-HCl (pH=8), 1% Nonident P-40 substitute, 0.5% DOC, 0.1% SDS (all v/v)), to which Ethylenediaminetetraacetic acid (5 mM end concentration) and 1 mini tablet of ROCHE complete protease inhibitor per 10 mL was added immediately before use, by hand crushing the brain material with a hand pestle in a 1.5 mL reaction tube. Lysis was completed by 1 min sonification on ice with a Sartorius Labsonic M Sonificator at 20% amplitude with care to avoid heating the suspensions. Finally, Lysates were centrifuged for 15 min at 17,200×g and 4 °C. The SN was subsequently collected, transferred to a new 1.5 mL reaction tube, flash frozen in liquid N_2 and stored at -80 °C until use.

Microarray Binding Assay

µSPOT slides were blocked by incubation with 2.5 mL 2% (w/v) IGG free bovine serum albumin (BSA) in PBS for 60 min at ~50 rpm and RT. Afterwards, slides were incubated with gephE or FL-geph at the desired concentration in 0.1% BSA in 1 × PBS for 30 min before slides were washed with 6×2.5 mL 1×PBS for 5 min. To label proteins for detection, the slides were incubated with 2.5 mL of a 1:10,000 diluted primary antibody (anti-gephyrin (3B11, SynapticSystems) in 0.1% BSA in 1×PBS for 30 min, after which the slides were washed with 6×2.5 mL 1×PBS for 5 min. Afterwards, the slides were incubated with a secondary HRP-coupled Anti-mouse antibody (31430, Invitrogen) in 0.1% BSA in 1×PBS for 30 min, after which the slides were washed with 6×2.5 mL 1×PBS for 5 min. Peptide binding was detected through chemiluminescent detection (Lowest Sensitivity, 10s exposure time) after application of 200 µL of SuperSignal West Femto Maximum Sensitive Substrate (Thermo Scientific) per slide using a c400 imaging system (Azure). Fluorescent detection (Cy3 channel, 700 V PMT, 25 µm pixel size, L5 latitude) as shown in Supplementary Figure 2 A was done using a Typhoon FLA 7000 scanner-based detection system (GE Healthcare).

Binding assays using fluorescently labelled gephyrin were performed similarly, without antibody staining. Peptide binding was detected through fluorescence detection (60 µm resolution, 10s exposure time) on a c400 imaging system (Azure).

For on-chip peptide competition assays, gephE was preincubated with either peptide **1a** or **1b** in 0.1% BSA in PBS for 30 min on ice before being put on an array slide.

Binding intensities were evaluated using FIJI including the Microarray Profile addon (OptiNav). After background subtraction of the mean greyscale value of the microarray surface surrounding the spots, raw greyscale intensities for each position were obtained for the left and right side of the internal duplicate on each microarray slide. The standard deviation (STDEV) between both sides was obtained using formula (1).

$$STDEV = \sqrt{\frac{\sum(x - \bar{x})}{n}} \qquad (1)$$

with

n The total number of data points
$\bar{x}$ The mean intensity value

Afterwards, the raw spot intensities and corresponding STDEVs were normalized to the most prominent spot within the region of interest on the array. Data normalization for representation of a heatmap as in Figure 5 A, B, C was performed lane-wise, while normalization as in Supplementary Figure 2 B was performed to the average of all 15 wildtype-sequence peptides due to the comparably low spot intensity (Supplementary Figure 2 A).

Temperature Related Intensity Change (TRIC) Assays

An assay buffer consisting of 1× PBS (pH 7.4), 2 mM L-Glutathione reduced (GSH) and 0.1 % Pluronic F127 was used for all experiments. Before the measurements, all peptides were solubilized in assay buffer to an end concentration of 500-700 µM. The unlabelled control peptide NND1 was dissolved in the same buffer to a concentration of 4 µM. A HAMILTON STARlet system was used for liquid handling.

For single dose measurements, peptides were first diluted from the stock solution into assay buffer to a 1:1 dilution in a conventional 384-microwell plate. In a second step, peptides were

mixed with the preincubated (1h at 4°C) target complex-containing solution consisting of gephE and NN1D-Alexa647, resulting in a final concentration of 20 nM and 10 nM respectively. This step took place in a Dianthus 384-microwell plate, in which the measurements were performed.

For 12-point affinity measurements, peptide solutions were first pre-diluted 1:1 in assay buffer, from which 0.5-fold dilutions were prepared in assay buffer in a standard 384-well plate. 15 µl of these dilutions were then mixed with 5 µl of target complex-containing assay buffer in a Dianthus 384-well plate, resulting in a final concentration of 5 nM gephE-NN1D-Alexa647 complex.

After the Dianthus 384-well microplates were loaded with the peptide and gephyrin-NN1D-Alexa647 complex, they were equilibrated for 16h at 4 °C and subsequently centrifuged for 30 sec at 1000×g immediately before starting the measurement in the Dianthus NT.23PicoDuo. The system was set to 25°C as set temperature. The samples were first measured for 1 sec without heating and for 5 sec with the IR-laser turned on. The two optical systems in Dianthus were used in parallel, resulting in an overall measurement time of ~30 min per plate. Measured fluorescence values collected are displayed as relative fluorescence, where the fluorescence obtained at ambient temperature is normalized to one, and as normalized fluorescence (F_{norm}) which describes the ratio between fluorescence values (F1) after and the fluorescence values (F0) prior to IR laser activation and is typically given in ‰. K_i values were obtained by applying a Hill-fit to a plot of F_{norm} vs. ligand concentration to determine an EC_{50} value, which was subsequently used to calculate the corresponding K_i value (Formula 2 and 3).

$$K_i = \frac{K_d}{2-\gamma} \cdot \left(\frac{EC_{50}}{\frac{[T]_t}{\gamma} - \frac{K_d}{2-\gamma} - \frac{[C]_t}{2}} - \gamma \right) \tag{2}$$

with

$$\gamma = \frac{[T]_t + [C]_t + K_d - \sqrt{([T]_t + [C]_t + K_d)^2 - 4[T]_t[C]_t}}{2[C]_t} \tag{3}$$

and

$[T]_t$	The total final concentration of the unlabelled target (gephE) in the assay
$[C]_t$	The total final concentration of fluorescent tracer (NN1D) in the assay that forms a complex with the target and is replaced by an unlabelled peptide ligand
K_d	The K_d between the fluorescent tracer and the target from a direct binding affinity measurement
EC_{50}	The EC_{50} obtained from titrating an unlabelled peptide ligand against the preformed complex of target and tracer

To provide a measure for the robustness of the dose response fits, the signal to noise ratio (S/N) was calculated from the signal amplitude of each fit and the residual values of all datapoints (Formula 4).

$$S/N = \frac{Signal\ Amplitude}{STDEV(Residuals)} \tag{4}$$

In the case of single dose measurements, the area response including all measured datapoints of the TRIC traces was considered instead of the F_{norm} value at a given timepoint. Here, the average area between the TRIC curve of the peptide ligand and the respective reference (no competitor peptide) is considered. Analysis of single-dose assays starts with the assignment of reference groups. A reference group designates a group of wells that are

all referenced together. In other words, the ligands within one reference group are compared to the same designated set of reference wells (wells with target protein but without ligand added). Next, the area between the traces obtained from all wells containing a reference sample are compared to one another. That results in a number of $((n^2-n)/2)$ areas, where n denotes the number of references. From these area values the mean reference area for that reference group is calculated. This procedure is repeated for all reference groups to yield their respective reference areas. The hit threshold is calculated in the following way (Formula 5):

$$Hit\ Threshold = m + z \cdot MAD \tag{5}$$

with

m Median of all reference group areas in the assay
z User-defined Z-score factor (7 in this study)
MAD Median absolute deviation of all reference group areas in the assay

Next, for each ligand well (that contains ligand and labeled target) the area between the TRIC trace of that well to all reference wells in the same reference group is calculated and averaged to yield a mean signal area value for that well. If multiple wells were measured per ligand (in this screen a duplicate was measured) the area values per well are averaged to yield an average area per ligand. If this ligand-specific signal area exceeds the hit threshold, the ligand is considered a hit, provided that the software did not identify other issues like ligand-induced fluorescence quenching or aggregation. Furthermore, the area between individual repeats for each ligand, the ligand area, is calculated. This area will indicate whether a given ligand yields a reproducible signal. From all relevant areas for one ligand, reference area, signal area and ligand area the software calculates a signal quality value that is generally a very robust measure for the signal-to-noise that is obtained for a given ligand. This signal quality is calculated in the following way (Formula 6).

$$Signal\ Quality = \frac{\overline{Area_{Signal}}}{\frac{(\overline{Area_{Reference}} + \overline{Area_{Ligand}})}{2}} \tag{6}$$

Supplemental Figures

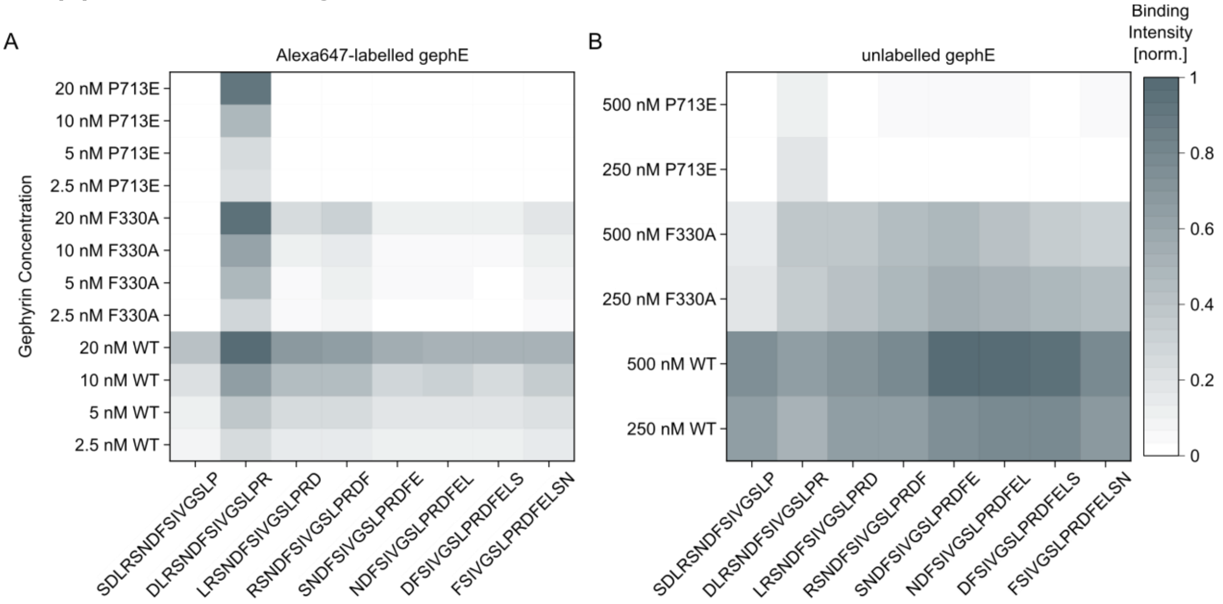

Supplemental Figure 1: Fluorescently labelled geph displays unspecific binding. Related to Figure 1. (A) Normalized binding intensities of Alexa647-labelled geph to GlyR β-derived peptides in µSPOT format. Note that for a certain peptide sequence (^{414}DLRSNDFSIVGSLPR^{428}), the binding intensity of fluorescently labelled WT geph is comparable to the binding intensity of two non-binding variants of gephE (F330A and P713E). (B) unlabelled gephE does not exhibit unspecific binding. Here, the relative binding intensity of WT geph is the highest overall, followed by F330A and P713E.

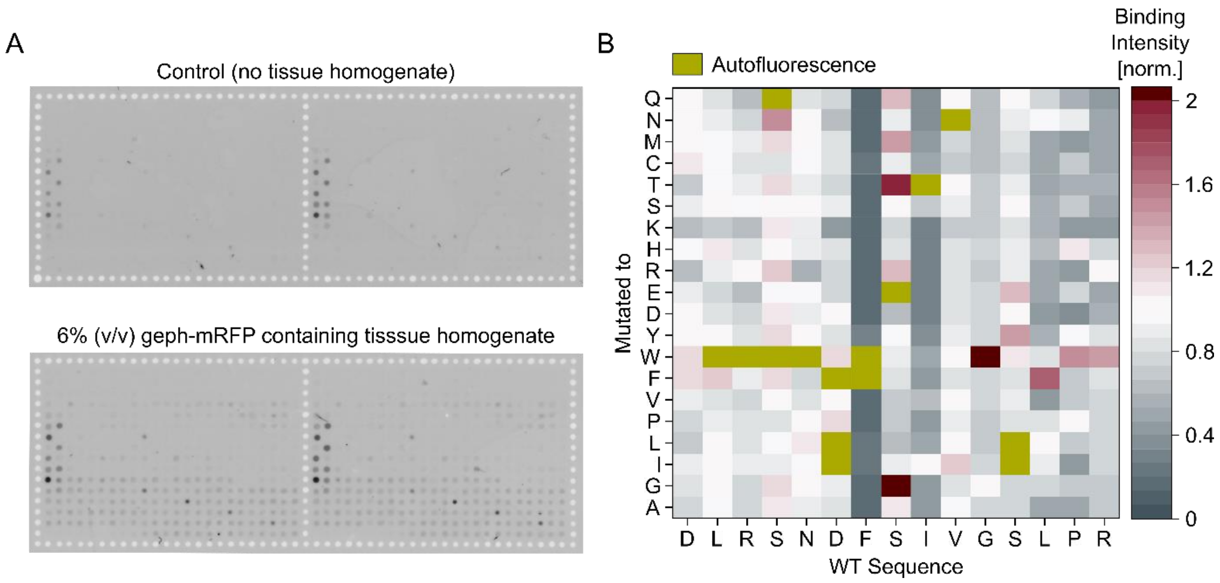

Supplemental Figure 2: Binding assays with mRFP-geph within tissue lysates from knock-in animals. Related to Figure 1 and 5. Tissue of mRFP-geph knock-in animals of 1 year of age provided by C Specht, IBENS, Paris was used directly on arrays. (A) raw readout of a microarray slide not treated with geph (upper panel) and treated with tissue lysate, containing geph fused to mRFP (lower panel). Note that autofluorescence of certain peptides in observed, while additional, geph-specific signal can be seen in the lower panel. (B) Shown are intensity values of point-mutated variants normalized to the corresponding wild-type sequence (GlyR β ^{414}DLRSNDFSIVGSLPR^{428}) displayed as a heatmap. Higher spot intensity corresponds to preferential binding, *vice versa*. Spots that showed autofluorescence in (A) are marked in green.

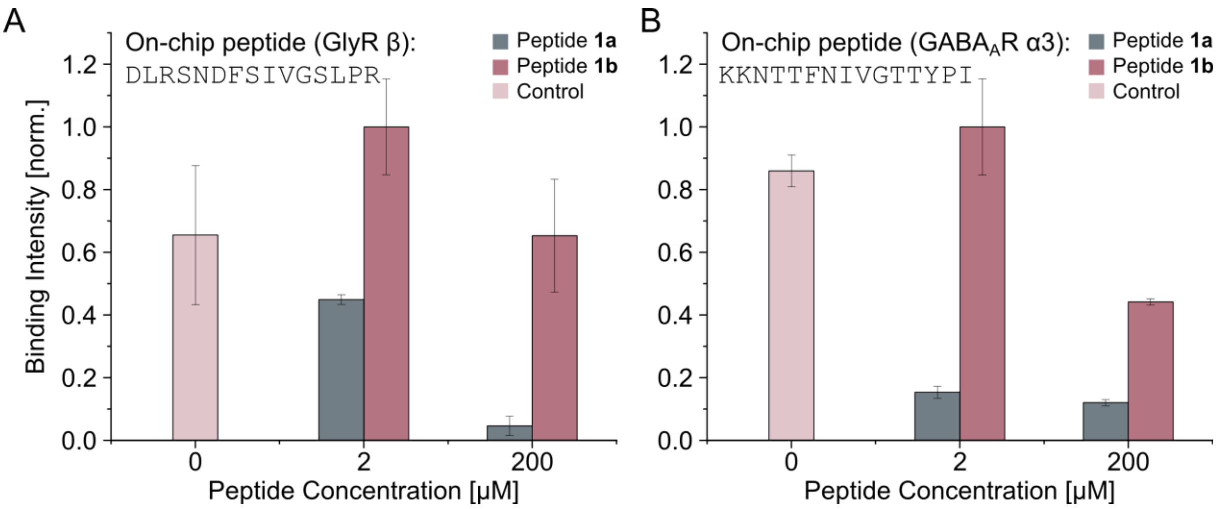

Supplemental Figure 3: In-situ on-chip peptide neutralization validates binding specificity. Related to Figure 1. gephE binding to on-chip peptides corresponding to either the GlyR β subunit (A) and the GABA$_A$R α3 subunit (B) was neutralized using peptides in solution, corresponding to either the GlyR β subunit (FSIVGSLPRDFELC, **1a**) and the GABA$_A$R α3 subunit (FNIVGTTY, **1b**). Note that to achieve neutralization of the GlyR β subunit, 200 µM of the corresponding peptide were necessary, while the GABA$_A$R subunits derived peptide could be neutralized using only 2 µM of the same peptide. Values are presented as n=2 with array internal STDEV.

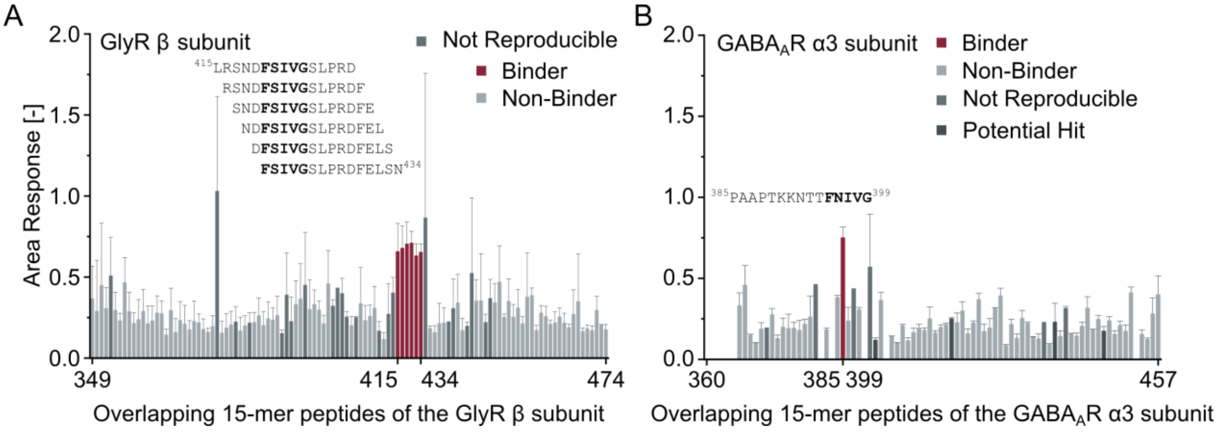

Supplemental Figure 4: Exhaustive screening of peptide libraries in solution using TRIC. Related to Figure 3. (B-C) Bar graph showing area values for each peptide measured (see Supplementary Table 3 and 4 for peptide sequences corresponding to the GlyR β (B) and GABA$_A$R α3 (C) subunit respectively and area values for each data point). Residues delimiting the peptides on-chip are represented on the x-axis. Hits are highlighted in dark red alongside corresponding peptide sequences with the respective binding motifs in bold. Note that only peptides bearing an ^{420}FSIVG^{424} or ^{395}FNIVG^{399} (GlyR β and GABA$_A$R α3 subunit respectively) were identified as binders. In addition to binders and non-binders, peptides classified as not reproducible or potential hits are highlighted. Values are presented as n=1-6 with corresponding STDEV if applicable.

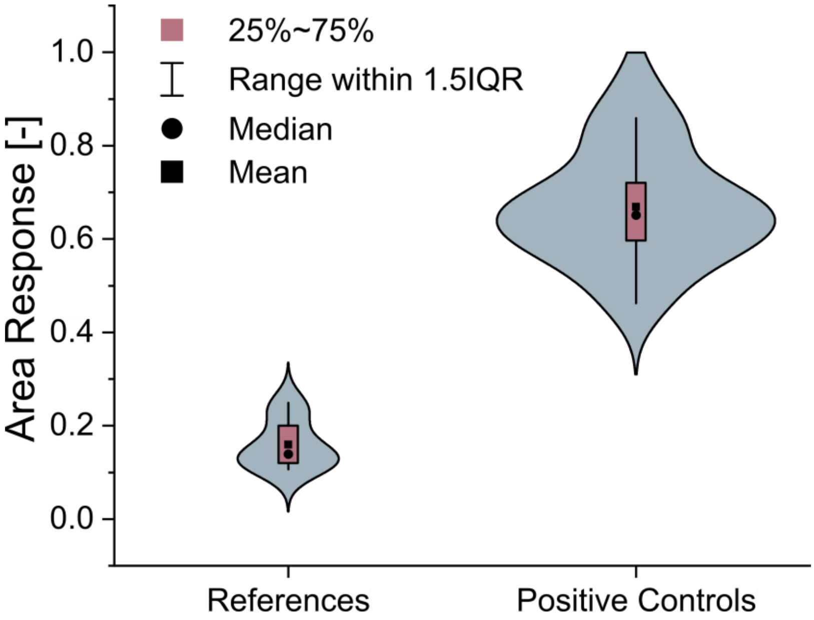

Supplemental Figure 5: Area response of reference and control measurements with unlabelled tracer. Related to Figure 3. Violin plot showing the distribution of reference measurements with no competitor peptide (n=8) and positive controls with unlabelled tracer (n=22) averaged over both 384-well plates used in single dose measurements.

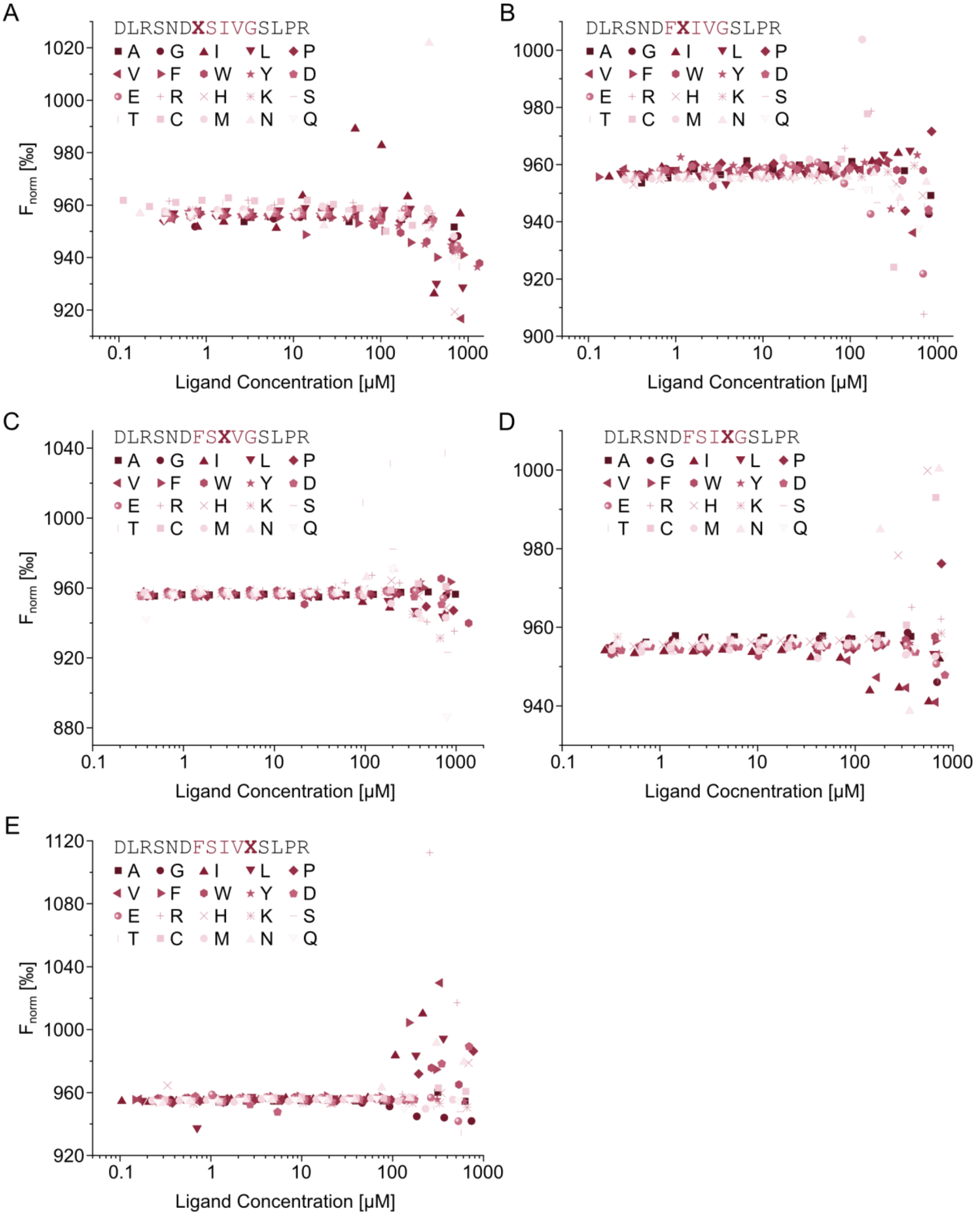

Supplemental Figure 6: Overview of all full positional scan TRIC data points. Related to Figure 5. Dose responses of 15mer peptides corresponding to variants of the core binding motif of the GlyR β subunit against geph were done in a high-throughput TRIC setup. The core binding motif is marked in red, with the variable position marked in bold. Refer to supplemental table 6 for starting concentrations and K_I values.

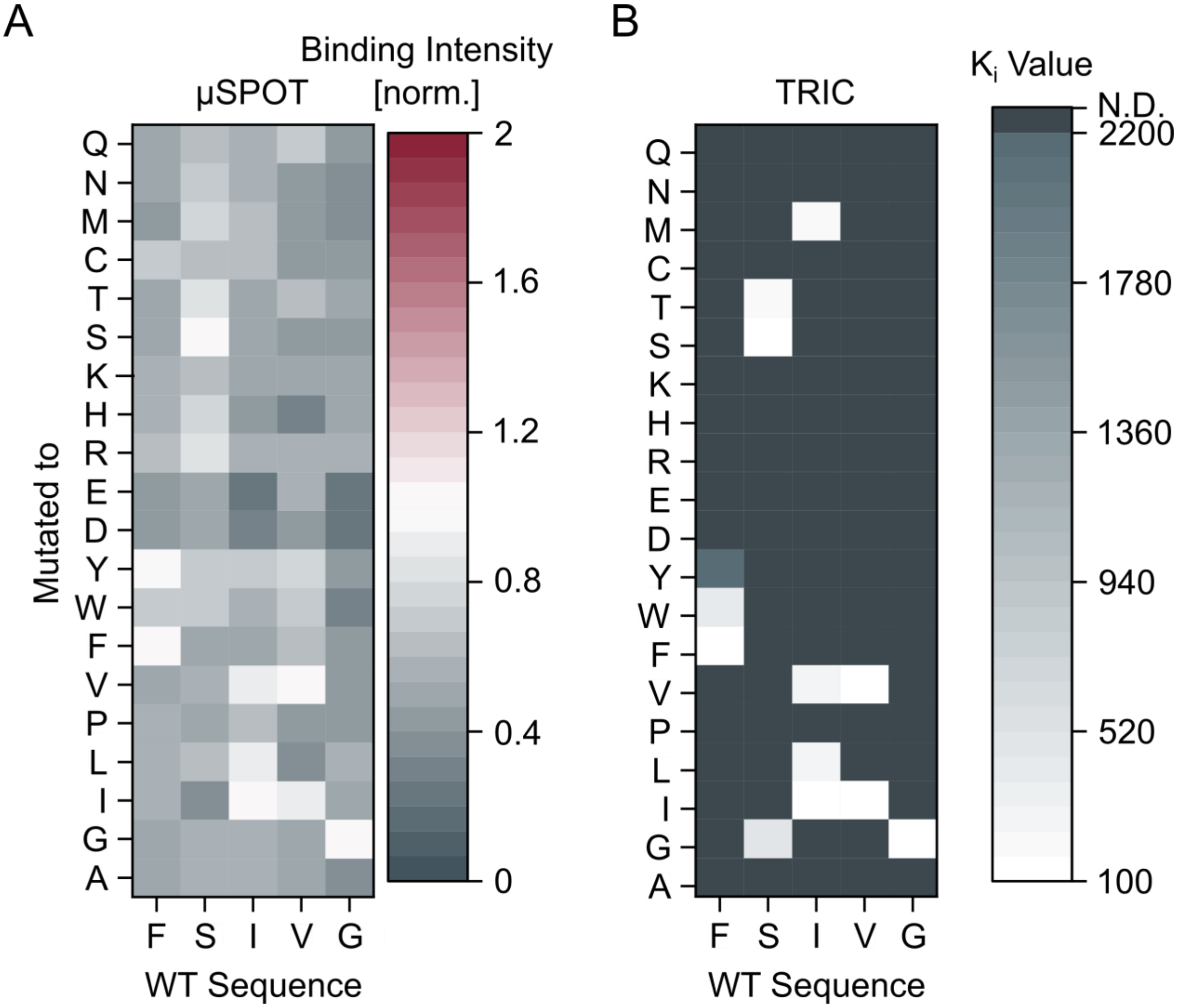

Supplemental Figure 7: Comparison of gephE/GlyR β subunit binding in µSPOT and TRIC. Related to Figure 5. (A) A full positional scanning library was probed with recombinant gephE in microarray format. Shown are intensity values of point-mutated variants normalized to the corresponding WT sequence (GlyR β ^{392}DLRSNDFSIVGSLPR^{406}) displayed as a heatmap. Higher spot intensity corresponds to preferential binding, *vice versa*. (B) The K_i values of a peptide library corresponding to the GlyR β subunit (^{414}DLRSNDFSIVGSLPR^{428}), varied to every proteogenic amino acid within the FSIVG core binding motif, were determined in a TRIC displacement approach. Note that for peptides exhibiting a close to WT binding intensity in µSPOT format a K_i value could be determined in TRIC format.

Supplemental Tables

Supplemental Table 1: Overlapping peptide library corresponding to GlyR β in µSpot. Related to Figure 1. Peptide sequences are given with their delimiting residue numbers alongside the relative binding intensity and STDEV obtained for 50 nM of gephE and 3% of mouse brain lysate containing native geph.

Peptide Name/Index	Peptide Sequence	Residues	gephE Binding Intensity [norm.]	STDEV	Native geph Binding Intensity [norm.]	STDEV
Glycine receptor subunit beta_001	VVQVMLNNPKRVEAEKARIA	349-368	0.017	0.003	0.081	0.006
Glycine receptor subunit beta_002	VMLNNPKRVEAEKARIAKAE	352-371	0.010	0.003	0.082	0.001
Glycine receptor subunit beta_003	NNPKRVEAEKARIAKAEQAD	355-374	0.023	0.003	0.109	0.005
Glycine receptor subunit beta_004	KRVEAEKARIAKAEQADGKG	358-377	0.052	0.007	0.117	0.003
Glycine receptor subunit beta_005	EAEKARIAKAEQADGKGGNA	361-380	0.024	0.004	0.113	0.003
Glycine receptor subunit beta_006	KARIAKAEQADGKGGNAAKK	364-383	0.040	0.003	0.091	0.010
Glycine receptor subunit beta_007	IAKAEQADGKGGNAAKKNTV	367-386	0.025	0.005	0.066	0.010
Glycine receptor subunit beta_008	AEQADGKGGNAAKKNTVNGT	370-389	0.023	0.009	0.065	0.012
Glycine receptor subunit beta_009	ADGKGGNAAKKNTVNGTGTP	373-392	0.015	0.003	0.067	0.017
Glycine receptor subunit beta_010	KGGNAAKKNTVNGTGTPVHI	376-395	0.024	0.004	0.077	0.009
Glycine receptor subunit beta_011	NAAKKNTVNGTGTPVHISTL	379-398	0.011	0.003	0.077	0.001
Glycine receptor subunit beta_012	KKNTVNGTGTPVHISTLQVG	382-401	0.023	0.003	0.085	0.000
Glycine receptor subunit beta_013	TVNGTGTPVHISTLQVGETR	385-404	0.008	0.000	0.087	0.003
Glycine receptor subunit beta_014	GTGTPVHISTLQVGETRCKK	388-407	0.044	0.000	0.068	0.012
Glycine receptor subunit beta_015	TPVHISTLQVGETRCKKVCT	391-410	0.013	0.001	0.060	0.006
Glycine receptor subunit beta_016	HISTLQVGETRCKKVCTSKS	394-413	0.042	0.006	0.042	0.004
Glycine receptor subunit beta_017	TLQVGETRCKKVCTSKSDLR	397-416	0.016	0.003	0.054	0.020
Glycine receptor subunit beta_018	VGETRCKKVCTSKSDLRSND	400-419	0.011	0.002	0.074	0.041
Glycine receptor subunit beta_019	TRCKKVCTSKSDLRSNDFSI	403-422	0.033	0.004	0.089	0.049
Glycine receptor subunit beta_020	KKVCTSKSDLRSNDFSIVGS	406-425	0.393	0.003	0.309	0.096
Glycine receptor subunit beta_021	CTSKSDLRSNDFSIVGSLPR	409-428	0.645	0.093	0.379	0.019
Glycine receptor subunit beta_022	KSDLRSNDFSIVGSLPRDFE	412-431	0.972	0.211	0.945	0.066
Glycine receptor subunit beta_023	LRSNDFSIVGSLPRDFELSN	415-434	1.000	0.265	1.000	0.085
Glycine receptor subunit beta_024	NDFSIVGSLPRDFELSNYDC	418-437	0.599	0.173	0.561	0.140
Glycine receptor subunit beta_025	SIVGSLPRDFELSNYDCYGK	421-440	0.291	0.143	0.301	0.019
Glycine receptor subunit beta_026	GSLPRDFELSNYDCYGKPIE	424-443	0.132	0.032	0.134	0.012
Glycine receptor subunit beta_027	PRDFELSNYDCYGKPIEVNN	427-446	0.428	0.016	0.521	0.039
Glycine receptor subunit beta_028	FELSNYDCYGKPIEVNNGLG	430-449	0.112	0.053	0.180	0.003
Glycine receptor subunit beta_029	SNYDCYGKPIEVNNGLGKPQ	433-452	0.216	0.003	0.204	0.004
Glycine receptor subunit beta_030	DCYGKPIEVNNGLGKPQAKN	436-455	0.049	0.017	0.093	0.006
Glycine receptor subunit beta_031	GKPIEVNNGLGKPQAKNKKP	439-458	0.036	0.005	0.069	0.018
Glycine receptor subunit beta_032	IEVNNGLGKPQAKNKKPPPA	442-461	0.009	0.001	0.075	0.004
Glycine receptor subunit beta_033	NNGLGKPQAKNKKPPPAKPV	445-464	0.055	0.002	0.041	0.009
Glycine receptor subunit beta_034	LGKPQAKNKKPPPAKPVIPT	448-467	0.022	0.001	0.061	0.009
Glycine receptor subunit beta_035	PQAKNKKPPPAKPVIPTAAK	451-470	0.012	0.002	0.087	0.002
Glycine receptor subunit beta_036	KNKKPPPAKPVIPTAAKRID	454-473	0.016	0.002	0.068	0.002
Glycine receptor subunit beta_037	KPPPAKPVIPTAAKRIDL	457-474	0.037	0.009	0.075	0.016

Supplemental Table 2: Overview of overlapping $GABA_A$R α3 peptide library in µSpot. Related to Figure 1. Peptide sequences are given with their delimiting residue numbers alongside the relative binding intensity and STDEV obtained for 20 nM of gephE and 6% of mouse brain lysate containing native gephyrin.

Peptide Name/Index	Peptide Sequence	Residues	gephE Binding Intensity [norm.]	STDEV	Native geph Binding Intensity [norm.]	STDEV
Gamma-aminobutyric acid receptor subunit alpha-3_001	NYFTKRSWAWEGKKVPEALE	360-379	0.062	0.005	0.048	0.022
Gamma-aminobutyric acid receptor subunit alpha-3_002	TKRSWAWEGKKVPEALEMKK	363-382	0.049	0.003	0.033	0.003
Gamma-aminobutyric acid receptor subunit alpha-3_003	SWAWEGKKVPEALEMKKKTP	366-385	0.064	0.034	0.118	0.009
Gamma-aminobutyric acid receptor subunit alpha-3_004	WEGKKVPEALEMKKKTPAAP	369-388	0.048	0.008	0.105	0.019
Gamma-aminobutyric acid receptor subunit alpha-3_005	KKVPEALEMKKKTPAAPTKK	372-391	0.038	0.007	0.081	0.023
Gamma-aminobutyric acid receptor subunit alpha-3_006	PEALEMKKKTPAAPTKKNTT	375-394	0.038	0.005	0.071	0.018
Gamma-aminobutyric acid receptor subunit alpha-3_007	LEMKKKTPAAPTKKNTTFNI	378-397	0.059	0.005	0.085	0.010
Gamma-aminobutyric acid receptor subunit alpha-3_008	KKKTPAAPTKKNTTFNIVGT	381-400	0.053	0.006	0.115	0.004
Gamma-aminobutyric acid receptor subunit alpha-3_009	TPAAPTKKNTTFNIVGTTYP	384-403	0.948	0.039	1.000	0.066
Gamma-aminobutyric acid receptor subunit alpha-3_010	APTKKNTTFNIVGTTYPINL	387-406	1.000	0.028	0.767	0.009
Gamma-aminobutyric acid receptor subunit alpha-3_011	KKNTTFNIVGTTYPINLAKD	390-409	0.443	0.018	0.243	0.006
Gamma-aminobutyric acid receptor subunit alpha-3_012	TTFNIVGTTYPINLAKDTEF	393-412	0.457	0.008	0.538	0.009
Gamma-aminobutyric acid receptor subunit alpha-3_013	NIVGTTYPINLAKDTEFSTI	396-415	0.044	0.006	0.114	0.042
Gamma-aminobutyric acid receptor subunit alpha-3_014	GTTYPINLAKDTEFSTISKS	399-418	0.030	0.004	0.098	0.058
Gamma-aminobutyric acid receptor subunit alpha-3_015	YPINLAKDTEFSTISKSAAA	402-421	0.039	0.005	0.114	0.045
Gamma-aminobutyric acid receptor subunit alpha-3_016	NLAKDTEFSTISKSAAAPSA	405-424	0.029	0.010	0.122	0.026
Gamma-aminobutyric acid receptor subunit alpha-3_017	KDTEFSTISKSAAAPSASST	408-427	0.020	0.005	0.136	0.010
Gamma-aminobutyric acid receptor subunit alpha-3_018	EFSTISKSAAAPSASSTPTA	411-430	0.028	0.003	0.127	0.000
Gamma-aminobutyric acid receptor subunit alpha-3_019	TISKSAAAPSASSTPTAIAS	414-433	0.024	0.001	0.116	0.004
Gamma-aminobutyric acid receptor subunit alpha-3_020	KSAAAPSASSTPTAIASPKA	417-436	0.030	0.001	0.103	0.031
Gamma-aminobutyric acid receptor subunit alpha-3_021	AAPSASSTPTAIASPKATYV	420-439	0.038	0.004	0.080	0.060
Gamma-aminobutyric acid receptor subunit alpha-3_022	SASSTPTAIASPKATYVQDS	423-442	0.022	0.001	0.075	0.063
Gamma-aminobutyric acid receptor subunit alpha-3_023	STPTAIASPKATYVQDSPAE	426-445	0.019	0.001	0.067	0.054
Gamma-aminobutyric acid receptor subunit alpha-3_024	TAIASPKATYVQDSPAETKT	429-448	0.018	0.004	0.060	0.032
Gamma-aminobutyric acid receptor subunit alpha-3_025	ASPKATYVQDSPAETKTYNS	432-451	0.020	0.006	0.050	0.016
Gamma-aminobutyric acid receptor subunit alpha-3_026	KATYVQDSPAETKTYNSVSK	435-454	0.070	0.001	0.059	0.022
Gamma-aminobutyric acid receptor subunit alpha-3_027	YVQDSPAETKTYNSVSKVDK	438-457	0.073	0.040	0.058	0.019

Supplemental Table 7: Purity of µSPOT peptides synthesized with C-terminal Rink amide linker. Related to Figure 1, 2, 3, 4 and 5. Concomitant cleavage and deprotection afforded soluble peptides which were analysed by LCMS. Protecting group by-products were not separated prior to analysis, and major impurities were identified by LCMS to be truncated sequences.

Peptide Sequence	M.W. [Da]	n	Purity [%]
Ac-SDLRSNDFSIVGSLP-NH_2	1604.82	2	19±1
Ac-DLRSNDFSIVGSLPR-NH_2	1673.88	2	39±2
Ac-LRSNDFSIVGSLPRD-NH_2	1673.88	2	58±2
Ac-RSNDFSIVGSLPRDF-NH_2	1707.87	2	45±2
Ac-KTPAAPTKKNTTFNI-NH_2	1629.92	2	71±13.5
Ac-TPAAPTKKNTTFNIV-NH_2	1600.89	2	86±1.5
Ac-PAAPTKKNTTFNIVG-NH_2	1556.87	2	88±3
Ac-AAPTKKNTTFNIVGT-NH_2	1560.86	2	84±5.5
Ac-SIVGSLPRDFELSNYDCYGK-NH_2	2261.08	2	51±2
Ac-GSLPRDFELSNYDCYGKPIE-NH_2	2301.07	2	44±8
Ac-PRDFELSNYDCYGKPIEVNN-NH_2	2372.07	1	73
Ac-FELSNYDCYGKPIEVNNGLG-NH_2	2230.04	2	47±0.5
Ac-KKKTPAAPTKKNTTFNIVGT-NH_2	2143.25	2	52±3.5
Ac-TPAAPTKKNTTFNIVGTTYP-NH_2	2120.13	2	37±3.5
Ac-APTKKNTTFNIVGTTYPINL-NH_2	2191.2	2	35±3.5
Ac-KKNTTFNIVGTTYPINLAKD-NH_2	2237.21	1	36

Supplemental References

DIKMANS, A., BEUTLING, U., SCHMEISSER, E., THIELE, S. & FRANK, R. 2006. SC2: a novel process for manufacturing multipurpose high-density chemical microarrays. *Qsar & Combinatorial Science,* 25**,** 1069-1080.

MARIC, H. M., KASARAGOD, V. B., HAUSRAT, T. J., KNEUSSEL, M., TRETTER, V., STRØMGAARD, K. & SCHINDELIN, H. 2014. Molecular basis of the alternative recruitment of GABA A versus glycine receptors through gephyrin. *Nature communications,* 5**,** 1-11.
